# Prevention-of-mother-to-child-transmission (PMTCT) program outcomes in South Africa in the pre-COVID and COVID eras

**DOI:** 10.1186/s12889-023-16214-5

**Published:** 2023-07-20

**Authors:** Keshena Naidoo, Monjurul Hoque, Somaya Buckus, Maariyah Hoque, Kathleen Jagernath

**Affiliations:** 1 Department of Family Medicine, University of KwaZulu-Natal, Durban, South Africa; 2 Kwadabeka Community Health Centre, KwaDabeka, South Africa; 3 South African College of Applied Psychology, Pretoria, South Africa

**Keywords:** Antenatal care, ART, MTCT, HIV, Pregnancy, South Africa

## Abstract

**Introduction:**

Two decades after implementing the Prevention of Mother to Child transmission (PMTCT) program, South Africa has still not managed to eliminate intrauterine mother-to-child (MTCT) HIV transmission. During the COVID pandemic access to maternal health services was reduced, potentially compromising the PMTCT program.

**Method:**

A retrospective record review was conducted at a midwife-run obstetric unit in a high HIV prevalence setting. Data on pregnant women who delivered between January 2019 and December 2020 were analysed to evaluate predictors for MTCT, and compare pre-COVID and COVID-era changes in maternal and infant HIV incidence and prevalence.

**Results:**

A total of 1660 women delivered at the facility over a 24-month period (Jan 2019-Dec 2020), of whom 92.8% enrolled for antenatal care in 2019 and 94.6% in 2020. A significantly greater proportion of women were aware of their HIV status before enrolling for antenatal care in the pre-COVID (2019) than COVID (2020) period (88% vs 40.2%; *p* < 0.05). There was a significant increase in new HIV infection after enrolling for antenatal care during the COVID period compared to pre-COVID period (120 vs 62 women, *p* < 0.05). There was also a significant increase in the HIV prevalence among women who delivered during the COVID period than in the pre-COVID era (43.5% compared to 35.8%, *p* < 0.05). However, more than 95% of HIV-positive women initiated ART in both periods. Overall, a total of thirteen infants tested HIV positive (2.1% MTCT rate), with no difference in MTCT between 2019 and 2020. Infants born to women on antiretroviral therapy (ART) were 93% less likely to have a positive PCR test than those whose mothers who were not on ART. (OR = 0.07, 95% CI 0.031:0.178, *p* < 0.05).

**Conclusion:**

The increase in maternal HIV incidence and prevalence during the COVID era suggest a lapse in HIV prevention strategies during the COVID pandemic. There is an urgent need to improve community test-and-treat campaigns among women of reproductive age in the community and increase access to HIV pre-exposure prophylaxis for pregnant women, especially during periods of health crises.

## Introduction

The incidence and prevalence of HIV among pregnant women in sub-Saharan African (SSA) countries remains high [1]. Estimated HIV prevalence rates are as high as 40% and new HIV infection during pregnancy is as high as 3.6/100 person-years in the SSA region [2]. The Prevention-of-Mother-to-Child Transmission (PMTCT) program aims to address the high prevalence of HIV among pregnant women and eliminate mother-to-child transmission (MTCT) [3]. In order to eliminate MTCT there needs to be less than 50 new child HIV infections per 100 000 live births, or less than 0.05% MTCT rate) [4]. However, it is unclear how the COVID pandemic has affected the PMTCT program and outcomes in the SSA region.

Since the implementation of the PMTCT program in South Africa in 2006 the intrauterine MTCT rate of HIV has steadily declined from 9.6% in 2008 to 2.7% in 2011, and to 0.9% in 2016 [3, 5, 6]. In contrast, studies in other African countries report MTCT rates as high as 8.1% in Ethiopia (95% confidence interval 7.3–12.9), while in Nigeria the MTCT rates are estimated at 2.74% (95% confidence interval: 2.48–2.99) [7, 8]. However, within South Africa there is great variability in the prevalence of in-utero MTCT between provinces, ranging between 168 and 325 cases per 100 000 live births [9–11]. The integration of HIV and antenatal care services has significantly improved coverage of the three key processes required to eliminate MTCT [12]. The three processes are for > 95% of pregnant women to receive antenatal care, > 95% test for HIV and for > 95% of HIV-positive women to receive antiretroviral therapy (ART) [9]. During the COVID pandemic delivery of these services was impaired.

The PMTCT program in South Africa, as in other low-to-middle income countries, is delivered primarily through midwife-run obstetric units (MOUs) that provide reproductive healthcare services to low-risk pregnant women [7, 10]. MOUs play a vital role in testing and caring for HIV-positive pregnant women, thereby reducing the risk for MTCT. Studies across the different provinces in South Africa report pre-COVID uptake of HIV testing to be as high as high as 99.8%, and over 95% of HIV positive pregnant women were on ART [8, 9]. Despite high levels of HIV testing and ART uptake, MTCT has still not been eliminated. Possible factors could be late HIV diagnosis and initiation of ART and poor ART adherence. T [8, 9, 13]. During the COVID pandemic maternal health services were further compromised when health services were reduced and communities were reluctant to attend health facilities for fear of contracting COVID [14]. South Africa experienced the first wave and the beginning of the second wave of the COVID-19 pandemic during the year 2020 [15]. Social and economic lockdowns during this period prevented many women from accessing family planning and antenatal health services, and reportedly increased HIV risk in pregnant women [16]. Studies in the sub-Saharan African region indicate that disruption of PMTCT services for just three months could have resulted in an estimated 50% increase in new HIV infections among children [17].

This study describes the implementation and outcomes of the PMTCT program before and during the COVID-19 pandemic at a MOU in KwaZulu-Natal, South Africa. The findings will thus provide some insights into the gaps in eliminating mother-to-child HIV transmission in the SSA region.

## Methods

### Study setting

The study was conducted at Kwadabeka community health centre in the eThekwini district of KwaZulu-Natal, South Africa. The KwaZulu-Natal province (KZN) in South Africa is noted to have the highest HIV prevalence nationally (estimated at 41.1%) [18]. Previous studies in the study setting estimated the HIV prevalence among pregnant women at 44% [11]. The facility provides primary care services to a predominantly black African population in the north-west outskirts of eThekwini district. The mostly rural community of approximately 100 000 people are of low socio-economic standing living in mainly informal and formal types of dwellings and having strong cultural ties with the rural people of KZN and Eastern Cape Provinces.

The annual headcount at the community health centre is approximately 200 000 and includes 5 000 visits to the MOU. The facility provides the only 24-h midwife-run obstetric unit in the area and conducts approximately 700 normal vaginal deliveries annually [11]. The MOU is staffed by professional midwives and nurses and managed by an operational Nursing Manager. High-risk pregnancies and obstetric emergencies are referred to the local district hospital, a 30-min drive away.

### Study design

A retrospective audit was conducted of all women who delivered at a MOU over a 24-month period (January 2019-December 2020). Total population sampling was conducted and all records during the study period were included in the analysis. The primary study outcomes of interest included a comparison of antenatal care uptake, HIV testing and treatment outcomes and positive infant PCR tests in the pre-COVID and COVID eras.

### Data collection and analysis

Data were extracted from facility records by two trained research assistants between September and October 2021 and captured using a data collection tool that was adapted from a previous study [11]. Data were collected for the period January 2019 to December 2020. Although there were no COVID cases reported in January and February 2020 in South Africa, the data for the two months were included in the COVID era as part of the calendar year. Less than 100 women delivered during this period and it is unlikely that the patient profile is significantly affected.

Data collected included socio-demographic characteristics of patients, obstetric history, HIV screening and testing and antiretroviral therapy. The research assistants were orientated regarding the study protocol and research instruments prior to data collection. All data was anonymised. The data were captured onto an Excel spreadsheet and then imported into SPSS 22.0 for analysis.

Sociodemographic and clinical data for women in the pre-COVID and COVID periods were analysed using descriptive summary measures and compared using Pearson chi-square (χ2) test with *p*-values. The demographic and clinical exposure variables that had significant differences for the outcome variable (PCR test positive) were entered into a logistic regression analysis. The results of the regression analysis are expressed in adjusted odds ratios (OR) for binary outcomes, with corresponding two-sided 95% confidence intervals (95% CI), and associated *P* values. *P* values < 0.05 were considered significant.

The final outcome was the intrauterine MTCT rate (PCR test positive among HIV-exposed infants) during the study period and associated factors.

### Ethical considerations

Ethical approval and waiver of informed consent was obtained from the Umgungundlovu Health Ethics Review Board (Reference no. UHERB 015/2020) as this was a retrospective chart review. Permission was also granted from the Research Committee of the KZN Department of Health and facility management. All the methods and procedures carried out in this study were in accordance with relevant guidelines and regulation.

## Results

There were 1563 women who enrolled for antenatal care, and 1660 women who delivered at the MOU during the study period. Some women who delivered at the MOU were unbooked or attended other health providers for antenatal care, and others delivered outside the study period, hence the discordance between the number of antenatal clinic attendees and the number of women who delivered.

### Antenatal HIV screening and treatment

Data extracted from the facility records documented that 792 pregnant women were enrolled for antenatal care in 2019 and 771 women in 2020.

The results of HIV screening and testing of antenatal clinic attendees at the MOU for each year are tabulated in Table 1 below.

**Table 1:** Antenatal HIV screening and testing in pre-COVID (2019) and COVID (2020) periods

HIV Screening and testing	Pre-COVID (2019)	During COVID (2020)	*P* value
	**Frequency (%)**	**Frequency (%)**	
	***N* = 792**	***N* = 771**	
**HIV status known prior to enrolling at ANC**			
**• Yes**	697 (88.0)	310 (40.2)	
**• No**	95 (12.0)	461 (59.8)	< 0.05
**HIV status prior to booking**			
**• Positive**	210 (25.5)	219 (28.4)	
**• Negative**	487 (61.5)	91 (11.8)	< 0.05
**• Unknown**	95 (12.0)	461 (59.8)	
**Antenatal HIV screening & testing**			
**Total HIV positive**	272 (34.3)	339 (44.0)	< 0.05
**• + ve prior to 1**^**st**^** ANC visit**	*210 (25.5)*	*219 (28.4)*	
**• + ve at 1**^**st**^** visit**	*41 (5.2)*	*88 (11.4)*	
**• + ve fter 12 week visit**	*21 (2.7)*	*32 (4.2)*	
**• HIV negative**	520 (65.7)	432 (56.0)	
**Uptake of antiretroviral therapy among HIV-positive women**			
**Uptake of ART among antenatal clinic attendees**	^a^*n* = 272	^a^*n* = 339	
	230 (84.5%)	322 (94.9%)	< 0.05

^a^HIV-positive women only

### HIV screening at first visit

There was a significant decrease in the proportion of women enrolling for antenatal care who knew their HIV status during the COVID pandemic as compared to the pre-COVID period (88.0% versus 40.2%, *p* < 0.0001). In addition, significantly more women were newly diagnosed with HIV when enrolling for antenatal care during the COVID pandemic compared to the pre-COVID period at the first visit (11.4% vs 5.2%; *p* < 0.05).

### HIV screening 12 weeks after booking

Upon retesting, a further 21 and 32 women were diagnosed with HIV infection in 2019 and 2020 respectively, all of whom commenced ART. There was a significant increase in the HIV incidence during the COVID period than in the period prior to COVID. (4.2% vs 2.7%; *p* < 0.05).

### Antenatal HIV prevalence and ART uptake

An overall increase in HIV prevalence was noted among the antenatal clinic attendees from pre-COVID to during the COVID era (34.3% to 44.0%, *p* < 0.01). The uptake of ART in both the pre-COVID and COVID periods was sub-optimal and did not meet the WHO recommendation of 95%. However, there was a notable improvement in ART uptake among women attending the facility during the COVID period (94.9% vs 84.5%; *p* < 0.05).

### Characteristics of women who delivered at the MOU

The obstetric details of women delivered at the MOU are displayed in Table 2 below. Of the 1660 women who delivered in the study period, 819 women delivered in 2019 and 841in the year 2020.

**Table 2:** Obstetric details of women that delivered at the facility between January 2019 and December 2020

Variables	Pre-COVID	COVID	Combined Number	*P* value
	**2019 (*N* = 819)**	**2020 (*N* = 841)**	**(*N* = 1660)**	
	**Number (%)**	**Number (%)**		
**Age distribution in years**				
** < 20**	117 (14.3)	125 (14.9)	242 (14.6%)	
** 20—24**	243 (29.7)	231 (27.4)	474 (28.5%)	
** 25–29**	234 (28.6)	241 (28.7)	475 (28.6%)	
** 30- 34**	164 (20.0)	167 (19.9)	331 (20.0%)	**0.7**
**35–39**	56 (6.8)	67 (8.0)	123 (7.4%)	
** > 40**	5 (0.6)	10 (1.2)	15 (0.9%)	
**Parity**				
** Nil parity (no previous deliveries)**	253 (30.9)	240 (28.6)	493 (29.7%)	
** 2-3 deliveries**	292 (35.6)	288 (34.2)	580 (34.9%)	
** 4-5 deliveries **	156 (19.0)	205 (24.4)	361 (21.7%)	** < 0.05**
** > 5**	118(14.5)	108 (12.9)	226 (13.6%)	
**Antenatal (ANC) booking**				
** • Yes**	760 (92.8)	796 (94.6)	1556 (93.7)	**0.42**
** • No**	59 (7.2)	45 (5.4)	104 (6.3)	
**Number of antenatal care visits**				
** 3-4**	262 (32.0)	251 (29.9)	513 (30.9%)	
** 5-7**	313 (38.2)	357 (42.4)	670 (40.4%)	**0.21**
** > 8**	185 (22.6)	188 (22.3)	373 (22.5%)	
** Un-booked (o visit)**	59 (7.2)	45 (5.4)	104 (6.3%)	
**ANC booking < 20 weeks gestational age**				
** • Yes**	420 (51.3)	435 (51.7)	855 (51.5%)	
** • No**	340 (41.5)	361 (42.9)	701(48.5%)	**0.33**
** • Unbooked**	59 (7.2)	45 (5.4)	104 (6.3)	
**Gestational age in weeks**				
** < 32**	17 (2.1)	28 (3.3)	45 (2.7%)	
** 32–36**	92 (11.2)	115 (13.7)	207 (12.5%)	**0.08**
** > 36**	710 (86.7)	698 (82.9)	1408 (84.8%)	
**HIV status**				
• HIV Positive	293 (35.8)	365 (43.4)	658 (39.6)	
➢ Diagnosed before delivery	*272 (33.2)*	*339 (40.3)*	*611 (36.8)*	** < 0.05**
➢ Diagnosed at delivery	*21 (2.6)*	*26 (3.1)*	*47 (2.8)*	
• Negative	526 (64.2)	476 (56.6)	1002 (60.3)	
**ART uptake**				
• HIV-positive	293	365	658	
• On ART	*287 (98.1)*	*353 (96.7)*	*640 (97.3)*	**0.4**
• Not on ART	*6 (1.9)*	*12 (3.3)*	*18 (2.7)*	
• HIV-negative	526	476	1002	

There were no significant differences in the ages of women who delivered in the pre-COVID and COVID periods. Women who delivered at the MOU during the study period ranged in age from 14 to 44 years old. There were 28.5% of women aged 20–24-years-old, 28.6% aged 25–29 years-old, 20% were 30–34 years-old and less than 15% were younger than 20-years-old. Few women who delivered were older than 40-years-old (0.9%) or 30–34-year-old (7.4%).

Almost a third of the women were nulliparous (had no previous deliveries). During the COVID period there were significantly more women with 3-4 previous deliveries and significantly fewer women with one to two deliveries previously who delivered. The proportion of women who had more than five previous deliveries (grand multiparous) were 14.5% and 12.9% in 2019 and 2020 respectively.

There was a non-significant increase in the proportion of women who delivered at the facility that also enrolled for antenatal care in the COVID compared to pre-COVID period, (94.6% in 2020 and 92.8% in 2019). In both years the enrolment for antenatal care was below the 95% target recommended to achieve elimination of MTCT. Also, only 51.5% of the overall number of patients had enrolled for antenatal care before 20 weeks gestational age.

Majority of women (86.7% and 82.9%) delivered at term. There was a non-significant increase in the number of pre-term (< 32 weeks and 32–36 weeks gestational age) deliveries during the COVID period.

At the time of delivery all unbooked and HIV-negative patients were offered free HIV testing. After testing, there were 21 (2.6%) and 26 (3.0%) new HIV-positive women identified in 2019 and 2020 respectively, all of whom enrolled onto ART. There was a significantly higher HIV prevalence at delivery in the COVID than in the pre-COVID period (43.5% compared to 35.8%, *p* < 0.05). However, the uptake of ART was good in both years (98.1% vs 96.7%, *p* = 0.4).

### Birth outcomes of HIV-positive women

Routine HIV testing of all HIV-exposed infants is conducted using a polymerase chain reaction (PCR) test. A positive test results indicates intrauterine MTCT. The outcomes for infant testing are outlined in Table 3.

**Table 3:** Birth outcomes of HIV-positive women

Variable	Pre-COVID	During COVID	TOTAL	*P* value
	**-2019**	**-2020**	**Frequency (%)**	
	**Frequency (%)**	**Frequency (%)**		
**Birth outcomes of HIV-positive women**	*N* = 293	*N* = 365	*N* = 658	
** •** number of HIV-positive women with live births	**•***279 (95.2)*	**•***345 (94.5)*	**•** 624 (94.8)	< 0.05
** •** number of women without live births	**•***14 (4.8)*	**•***20 (5.5)*	**•** 34 (5.2)	
**PCR testing of HIV-exposed infants**	*N* = 279	*N* = 345	*N* = 624	
** •Yes**	234 (83.9)	297 (86.1)	531 (85.1)	
* - Positive*	*6 (2.2)*	*7 (2.0)*	13 (2.1)	> 0.05
* - Negative*	*228 (81.8)*	*292 (98.0)*	520 (97.9)	
** •No**	43 (15.4)	45 (13.0)	88 (14.1)	
** •Not documented**	2 (0.7)	3 (0.9)	5 (0.8)	

There were 279 HIV-positive women in 2019 who delivered live infants in 2019 and 345 in 2020. The proportion of live births among HIV-positive women was significantly lower during COVID.

PCR testing was only conducted on 83.9% of HIV-exposed infants in 2019 and 86.1% in 2020 (Table 3).

There were six (6) positive PCR results recorded among the HIV-exposed infants in 2019 and seven (7) in 2020. The MTCT rate for the study period was thus calculated as 2.1% (95% CI: 1.5–2.5) with no significant difference in between 2019 (2.2%) and 2020 (2.0%) (*p* > 0.05) (Table 3). This translates to 2150 HIV-infected infants per 100 000 deliveries of HIV positive women in 2019 and 2028 in 2020.

Further analysis was conducted with cross-table analysis with Pearson’s Chi-square (X2) and *p* values to identify significant maternal characteristics associated with MTCT of HIV over the 24-month study period (Table 4).

**Table 4:** Cross-table analysis of maternal features and positive PCR test result as an outcome variable

VARIABLE	Number of women who delivered (%)	Number of HIV-exposed infants with positive PCR test (%)	Chi-square (X^2^) value	*P* value
	***N* = 1660**	***N* = 13**		
**Maternal age at delivery in years**				
** < 20**	242 (14.6%)	1 (7.7)		
**20–24**	474 (28.5%)	2 (15.4)		
**25–29**	475 (28.6%)	3 (23.1)		
**30–34**	331 (20.0%)	3 (23.1)	**9.72**	0.032
**35–39**	123 (7.4%)	3 (23.1)		
** > 40**	15 (0.9%)	1 (7.7.)		
**Parity**				
**Nil**	493 (29.7%)	4 (27.3)		
**1**-**2**	580 (34.9%)	5 (39.9)	**1.17**	0.032
**3**-**4**	361 (21.7%)	2 (22.7)		
** > 5**	226 (13.6%)	2 (12.1)		
**Number of maternal antenatal visits**				
** • None**	104 (6.3)	1 (7.7)		
• **1**-**3**	513 (30.9%)	4 (30.8)	0.28	0.973
• **4**-**7**	670 (40.4%)	5 (38.5)		
• **> 7**	373 (22.5%)	3 (23.0)		
**Mother enrolled before 20 weeks gestation for antenatal care**				
• **Yes**	855 (51.5%)	5 (38.5)	3.63	< 0.05
• **No**	805 (48.5%)	8 (61.5)		
**Gestational age in at delivery**				
** < 32 weeks**	45 (2.7%)	0 (0.0)		
**32–36 weeks**	207 (12.5%)	1 (7.7)	2.74	0.254
** > 36 weeks**	1408 (84.8%)	12 (92,3)		
**On ART (*n* = 658)**	***N* = 658**			
• **Yes**	*640 (97.3)*	10 (77.0)	9.26	< 0.05
• **No**	*18 (2.7)*	3 (23.0)		

There were no significant associations noted between a positive PCR results and the number of antenatal visits or gestational age at delivery. However, significant positive PCR results was associated with maternal age, parity, antenatal enrolment before 20 weeks and ART (*p* < 0.05). Logistic regression was conducted for the variable of maternal ART only. These significant variables were entered into a step by step (backward) multivariate logistic regression analysis to identify predictors for the outcome variable.

Logistic regression output (Table 5) showed that babies born to women who were on ART was 93% less likely to test positive for HIV than those infants whose mothers were not on ART (OR = 0.07, 95% CI 0.031:0.178, *p* < 0.05).

**Table 5:** Final output of the logistic regression of the mother to child transmission of HIV among a cohort of women managed at the MOU from 2019 to 2020

Variables	Significance (*P*-value)	Adjusted odds ratio (OR))	95% CI for OR	
			**Lower**	**Upper**
On ART	0.000			
On ART (Yes)	0.000	0.074	0.031	0.178
Constant	0.000	0.139		

Reference groups: not on ART

## Discussion

This study reports on the outcomes of the PMTCT program at a midwife-run obstetric unit in a high HIV-prevalence setting before and during the COVID-19 pandemic. Enrolment into antenatal care (ANC) in both the pre-COVID and COVID periods was less than 95% and did not meet one of the three key processes required to eliminate MTCT. In addition, HIV testing prior to enrolling for ANC was poor in both periods but significantly lower during the COVID period. A likely factor is the suspension of HIV voluntary counselling and testing services during the COVID pandemic. HIV self-testing would assist in improving testing rates especially during times of health crises. Pre-conception testing and uptake of ART would improve maternal and child outcomes. In addition, self-testing could pick up HIV incidence during pregnancy and enable women to request treatment early rather than wait for routine antenatal visits [19].

Unlike studies elsewhere in South Africa that reported no changes in PMTCT indicators in the first COVID wave, the findings of this study revealed an increase in HIV incidence among pregnant women [20]. As predicted by health analysts disruptions to health services such as condom supplies and peer education would make populations more susceptible to increases in HIV incidence [21].

In this study, a significantly greater proportion of women enrolling for antenatal care during the pandemic tested HIV positive, suggesting that HIV incidence may have increased during 2020 [17]. HIV incidence during pregnancy was also significantly higher during the COVID period. The increasing number of new HIV infections diagnosed among pregnant women during antenatal clinic visits and even at delivery is also alarming and requires further inquiry into HIV prevention strategies at MOUs. There is an evident need for HIV prevention strategies to be targeted in this population group, especially during periods of health service disruptions. Pre-exposure prophylaxis (PrEP) is an HIV prevention strategy recommended for people at high risk for HIV infection. The South African guidelines for PrEP follow the WHO recommendations in considering pregnant and breastfeeding women for PrEP [35, 36] However, pregnant HIV-negative women, who are a high-risk group, in South Africa have had limited access to PrEP thus far [22]. It is also imperative that MOUs continue to offer repeat HIV testing throughout pregnancy and in the postpartum period [23, 24].

Once enrolled for antenatal care more than 95% of women seen at this facility tested for HIV and accepted ART if positive. Pregnant women during the COVID period had significantly higher uptake of ART than in the pre-COVID period, and attained the target recommended. It is likely that lack of social and economic activity during COVID improved both attendance at ANC and uptake of ART.

Midwife-run obstetric units play an important role in offering HIV screening, prevention and treatment to a high-risk population. However, there is still a large number of women who only present to health facilities to deliver. It is unknown how many women attend traditional birth attendants. The recommendations for Basic Antenatal Care (BANC) are that pregnant women should book before 20 weeks gestation. However, in this cohort only 55% of patients booked before 20 weeks. Other studies in KZN also report that most women only book in the 2^nd^ trimester [25]. Women who book late may have a delayed diagnosis of HIV and thus delayed initiation of ART [9]. The findings of this study highlighted the association between late ANC enrolment and MTCT. Greater public health initiatives are required to improve the early booking rate of pregnant women, such as engaging community health workers in identifying and referring pregnant women in the community.

There was a significant increase in the HIV prevalence rate among both antenatal clinic attendees and women who delivered during the pandemic era. However the HV prevalence among ANC attendees is rate is lower than the prevalence reported in 2018 (44.3%) for the same health facility [11]. Further data is required to determine the factors for changes in HIV prevalence in the community served by the MOU.

The MTCT rate at birth was 2.1%, or 2 150 per 100 000 live births, which was significantly higher than reported in earlier studies (1.5% in 2017 from SA) [9]. Previous studies reported a positive association between higher MTCT rates at birth and suboptimal PMTCT service coverage, and thus recommended early treatment of maternal HIV as a way of reducing vertical transmission [13]. A concerning issue was the non-availability of almost 15% of PCR results of HIV exposed babies in spite of blood samples sent to the hospital laboratory. The finding highlights the challenges of laboratory testing at primary care levels. Possible reasons for the missing results could be blood sample being lost in transit, inadequate sample, or samples being discarded at the laboratory. Evidence on point-of-care HIV testing for early infant diagnosis indicate that this is an accurate, feasible and acceptable method of diagnosing intrauterine MTCT [26]. However, this is not yet the standard of care in primary care facilities.

Intrauterine mother-to-child HIV transmission was positively associated with women who did not accept ART. Women who initiated ART were 93% (OR = 0.07) less likely to have MTCT than women not on ART. However, ART needs to be commenced at least 12 weeks before delivery to significantly reduce MTCT. In this study, although the uptake and adherence to ART during ANC met required levels the late diagnosis and initiation of ART may reduce the efficacy of the PMTCT programme. Further evidence is needed on factors for poor HIV testing prior to pregnancy and factors for poor ANC attendance.

### Strengths and limitations of this study

- Data were captured from a single centre and the results may not be applicable to other facilities.
- Women who delivered in the two months prior to the start of the COVID pandemic (January -February 2020) are included in the COVID era cohort.Data on viral load is not included hence the effect of viremia on MTCT cannot be commented on.

## Conclusion

Despite the increased prevalence of HIV among women who delivered during the COVID period there was no change in the MTCT rate. Maternal antiretroviral therapy significantly reduced the risk for MTCT. There is thus an urgent need for improved HIV testing and treatment among women of reproductive age in the community, including self-testing, during periods of health crises. The increase in HIV incidence among pregnant women during the COVID period also warrants increased access to PrEP for pregnant women.

## Data Availability

All data generated or analysed during this study are included in this published article. All data is available on request from the corresponding author.
